# Association Between Digit Ratio and Postoperative Nausea and Vomiting: A Single‐Center Prospective Cohort Study

**DOI:** 10.1155/anrp/8897870

**Published:** 2026-03-04

**Authors:** Yoshihiko Chiba, Yusuke Iizuka, Shinnosuke Ohama, Kyoko Chiba, Koichi Yoshinaga, Kyosuke Takahashi, Masamitsu Sanui

**Affiliations:** ^1^ Department of Anesthesiology and Critical Care Medicine, Saitama Medical Center, Jichi Medical University, Saitama-shi, Saitama, Japan, jichi.ac.jp; ^2^ Department of Anesthesiology and Critical Care Medicine, Jichi Medical University, Shimotsuke-shi, Tochigi, Japan, jichi.ac.jp; ^3^ Department of Anesthesiology, Tokyo Medical and Dental University, Bunkyo-ku, Tokyo, Japan, tmd.ac.jp

## Abstract

**Background:**

Postoperative nausea and vomiting (PONV) occurs even with preventive measures. Several reports suggest that PONV is associated with sex hormones. However, estimating individual hormone levels remains challenging. The digit ratio, the length ratio of the second to the fourth fingers, is a noninvasive predictor of hormone levels, with a ratio ≥ 1 being more feminine. We investigated whether digit ratio predicts PONV.

**Methods:**

This single‐center prospective study included patients aged > 18 years who underwent general anesthesia for scheduled surgery at the Jichi Medical University Saitama Medical Center. PONV prophylaxis was administered according to the number of risk factors defined by the well‐established Apfel simplified risk score (female, nonsmoking, history of motion sickness or PONV, and postoperative opioid usage). The primary endpoint was PONV incidence within 24 h postoperatively, stratified by the digit ratio. As a subgroup analysis, we analyzed the impact of the digit ratio on the incidence of PONV, which was further categorized according to sex, age, and type of surgery. Univariable and multivariable logistic regression analyses were also conducted to identify the risk factors associated with PONV.

**Results:**

Overall, 792 patients were included, with 19.1% having a digit ratio ≥ 1 and 80.9% < 1. Significant differences were observed in sex and age between the groups. PONV incidence within 24 h was 18.5% in the digit ratio ≥ 1 group and 18.7% in the digit ratio < 1 group, with no significant difference. Subgroup analyses by sex and younger age also showed no significant differences, except for a higher PONV incidence in abdominal surgery patients with digit ratio ≥ 1. Regression analysis identified the Apfel simplified risk score as a significant risk factor for PONV.

**Conclusions:**

Digit ratio was not associated with PONV in the overall patient population. However, an association was found in patients who underwent abdominal surgery, suggesting that digit ratio might be a risk predictor in this subgroup. Future studies should focus on larger sample sizes of patients undergoing high‐risk surgeries to validate these findings.

**Trial Registration:** University Hospital Medical Information Network Clinical Trials Registry (UMIN‐CTR); UMIN000048615

## 1. Introduction

Postoperative nausea and vomiting (PONV) is a postoperative patient discomfort factor similar to wound pain [1]. The incidence of PONV during general surgery is 30%, with an incidence of 80% in high‐risk patients [2]. In addition to being distasteful to the patient, PONV is associated with a longer time in the postanesthesia care unit [3] and increased healthcare costs [4].

Typical risk factors for PONV include the Apfel simplified risk score (female, nonsmoking, history of motion sickness or PONV, and postoperative opioid usage) [2, 5, 6], type of surgery [6, 7], use of inhaled anesthetics [6, 8], duration of anesthesia [6, 9], and younger age [6, 9]. However, PONV can occur even when preventive measures are taken based on these risk factors. Some individuals experience PONV, whereas others do not, even if they have the same risk factors and receive the same preventive measures. Therefore, a more precise scoring system and improved preventive measures are required.

Although sex is a strong risk factor for PONV, the underlying reason for this increased risk remains unclear. Several reports suggest that PONV is related to sex hormone–related events, such as the menstrual cycle [10–12] and menopause [13]. Estrogen, estradiol, and progesterone are associated with PONV [11]. These findings indicate that PONV is associated with sex hormone levels. The risk assessment for PONV may be more refined if sex hormone levels are considered in addition to sex binaries. However, it is difficult to estimate the levels of sex hormones in individual backgrounds. It is unknown whether there are any simple physical findings that can infer sex hormone levels. Therefore, we focused on finger length ratio, which could potentially be an indicator of sex hormones.

The digit ratio, which is the ratio of the length of the second to fourth fingers, is related to several hormonal levels, physical characteristics, and diseases [14–19]. A digit ratio of one or greater (≥ 1) is associated with femininity. Muller et al. suggested that the digit ratio may be related to risk and age at breast cancer onset [19]. Women with a digit ratio ≥ 1 are older at menopause [18] and have higher reproductive success than those with a digit ratio of less than one (< 1) [17]. The digit ratio is thought to be influenced by prenatal hormone exposure and is considered to be associated with the subsequent hormone balance [14, 18]. The digit ratio may be a noninvasive alternative predictor of sex hormones and is simple, safe, and inexpensive.

Assuming that a digit ratio ≥ 1 is considered more feminine, we hypothesized that patients with digit ratios ≥ 1 would have a higher incidence of PONV than those with digit ratios < 1. If the digit ratio could be identified as a risk factor for PONV, it would be possible to take more thorough measures against PONV, such as administering prophylaxis and changing anesthesia methods, rather than assuming a risk based only on the Apfel simplified risk score and taking measures. Consequently, this is socially significant because it can reduce postoperative patient discomfort and medical costs.

## 2. Methods

This single‐center prospective study was approved by the institutional review board of the Jichi Medical University Saitama Medical Center (IRB number: S22‐036, approved on August 1, 2022). The study was registered in the University Hospital Medical Information Network Clinical Trials Registry (UMIN‐CTR) before enrollment. Written and verbal informed consent was obtained from all participants, and an opt‐out option was made available. The study was conducted in accordance with the Declaration of Helsinki and applicable national ethical guidelines.

### 2.1. Patient

Patients who underwent surgery between August 11, 2022, and November 11, 2022, at the Jichi Medical University Saitama Medical Center were eligible for this study. Inclusion criteria were patients over 18 years of age undergoing general anesthesia for scheduled surgery, anesthesia time of 90 min or longer, and patients scheduled for extubation in the operating room. Exclusion criteria were patients < 18 years, emergency surgery, surgery with anesthesia expected to last less than 90 min, patients with dementia, patients with difficulty measuring finger length, surgery scheduled to be discharged within 24 h, and patients with difficulty being evaluated after 24 h.

### 2.2. Digit Ratio Measurement

**FIGURE 1:**
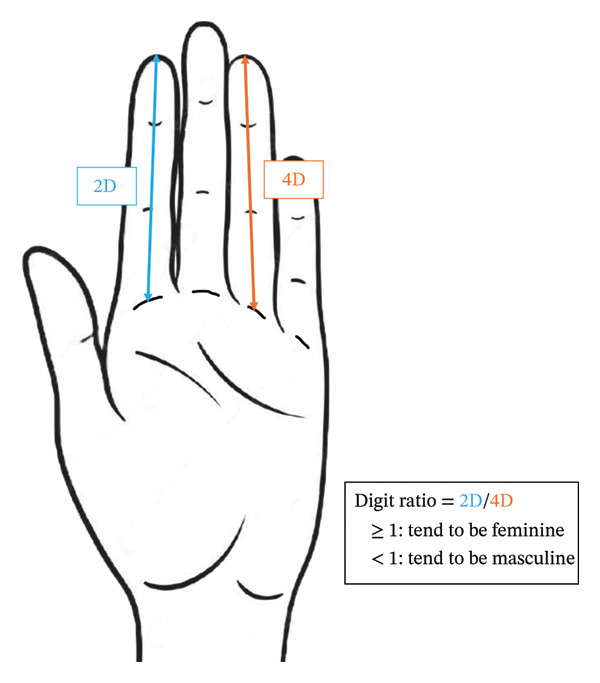
Measurement of digit ratio (2D:4D). This figure illustrates the process of measuring the digit ratio, which is defined as the ratio of the length of the second digit (2D, blue arrow) to that of the fourth digit (4D, orange arrow). The digit ratio was calculated by dividing the length of the index finger (2D) by that of the ring finger (4D). The measurements were conducted using digital calipers capable of measuring to 0.01 mm. This method involved measuring the interphalangeal crease of each finger to the tip with the hands laid flat on a table and the palms facing up. Each measurement was performed twice to ensure accuracy. A digit ratio of one or greater tended to be associated with more feminine traits, whereas a digit ratio of less than one tended to be associated with more masculine traits.

The digit ratio of the second (index) and fourth (ring) fingers of both hands was measured using digital calipers in the hospital room before surgery. The measurement method was based on previous studies [14–19], which measured the distance from the interphalangeal crease of the finger to the top of the finger (Figure 1). During the measurements, the participants’ hands were laid flat on a table with their palms facing up. The lengths of the second and fourth fingers of both hands were measured twice using a digital Vernier caliper capable of measuring to 0.01 mm. In this study, the digit ratio was defined as ≥ 1 if the second finger length on either hand was greater than or equal to the fourth finger length. Finger measurements were performed by only three trained persons who were confirmed to reliably perform the measurement method to avoid any variation. The doctors who performed the measurements were not involved in determining PONV prophylaxis.

### 2.3. Perioperative Management of Anesthesia and PONV

The choice of anesthesia method was entrusted to the anesthesiologist based on the patient’s preoperative information. Fentanyl, remifentanil, morphine, sevoflurane, desflurane, propofol, midazolam, remimazolam, and rocuronium were administered to induce and maintain anesthesia. The quantity of opioids administered was also determined at the discretion of the anesthesiologist, with the aim of providing adequate intraoperative and postoperative analgesia in consideration of the patient’s perioperative status.

We administered PONV prophylaxis commonly used at our institution, based largely on the consensus guidelines for PONV [20]. Preoperatively, the Apfel simplified risk score was used to identify the number of risk factors. Subsequently, two prophylaxis sessions were administered if one or two risk factors were present, and three or more prophylaxis sessions if three or more risk factors were present. Prophylaxis included a 5HT3 receptor antagonist (ondansetron or granisetron), total intravenous anesthesia with propofol, corticosteroids, and a dopamine antagonist (droperidol). The dosages and timing were determined according to guidelines [20].

### 2.4. Outcome

The primary endpoint was the incidence of PONV within 24 h after surgery in patients with digit ratios of ≥ 1 and < 1. Additionally, the usage of antiemetics in the wards and intensive care units (ICU) was examined. In a subgroup analysis, we analyzed the impact of the digit ratio on the incidence of PONV stratified by sex, age (< 50 years), and type of surgery. The analysis focused on types of surgery considered to have a high risk of PONV, including laparoscopic surgery, gynecologic surgery, and abdominal surgery (gynecologic, urologic, and intestinal surgeries involving the peritoneum or retroperitoneum) [20, 21]. Univariable and multivariable logistic regression analyses were used to examine the factors contributing to the incidence of PONV and to verify whether they were consistent with previous studies.

### 2.5. Data Collection

The assessment of preoperative PONV risk in patients was conducted by an assigned anesthesiologist using a standardized paper template. This template included fields for surgery date, patient name, patient ID, PONV risk factors based on the Apfel simplified risk score, and intraoperative prophylactic medications for PONV. If the information collected during preoperative assessment in the outpatient department was insufficient, the anesthesiologist obtained the necessary details from the patient before surgery.

Postoperative data on PONV and the use of antiemetic medications were evaluated from medical records. A case was defined as having PONV if it was noted in the chart at least once within the first 24 h after surgery. The presence of PONV was checked at least every 2–4 h in the general wards and every 2 h in the ICU. All data recorded in the charts, along with other patient information, such as sex, age, and physical information, were obtained as electronic data from the hospital’s information department after completion of all surgeries for the study subjects.

### 2.6. Sample Size Estimation and Statistical Analysis

PONV incidence at our hospital was approximately 20% when averaged for males and females. Before this study, we measured the digit ratio for 120 individuals, and the proportion of digit ratio ≥ 1: digit ratio < 1 was 1:4. Based on this value, we assumed that the incidence of PONV was 29% in the group with a digit ratio ≥ 1 and 18% in the group with a digit ratio < 1. A total of 750 subjects (150:600) were used to calculate the sample size, assuming an alpha error of 0.05 and a detection power of 0.8. With an expected dropout rate of 15%, the target number of patients was 882.

Categorical data are expressed as frequencies (%) and were evaluated using the chi‐square test or Fisher’s exact test. All continuous data are presented as the mean and SD for normally distributed variables, analyzed using Welch’s *t*‐test, and as the median and IQR for non‐normally distributed variables, analyzed using the Mann–Whitney *U*‐test. The risk factors and preventive measures for PONV were included in univariable and multivariable logistic regression analyses. The following explanatory variables of the regression model were clinically selected as possible risk factors for PONV: female sex, nonsmoking status, history of PONV or motion sickness, postoperative opioid usage, inhalation anesthetics, duration of anesthesia, age, type of surgery with associated risks, and use of prophylactic drugs. The types of surgeries considered risky included gynecologic surgery, laparoscopic surgery, cholecystectomy, and abdominal surgery. All statistical analyses were performed using EZR software (Saitama Medical Center, Jichi Medical University, Saitama, Japan). Statistical significance was set at *p* < 0.05.

## 3. Results

### 3.1. Preoperative Patient Characteristics

**FIGURE 2:**
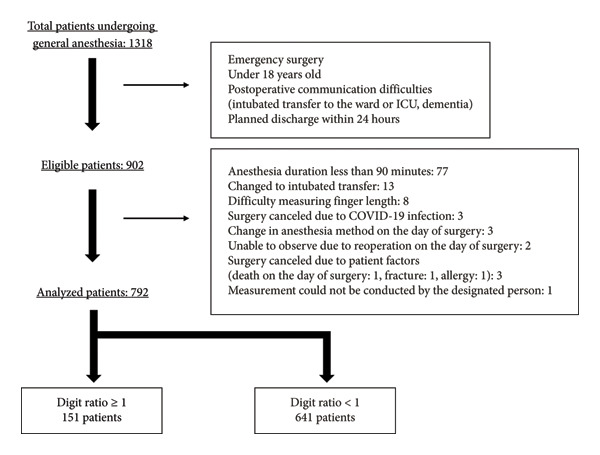
Flowchart of patient inclusion and analysis. ICU: intensive care unit.

From August 11, 2022, to November 11, 2022, 1318 patients received general anesthesia in the operating room. Of these, 902 were included in the study after excluding those who met the exclusion criteria. Among them, 792 were included in the analysis (Figure 2). Preoperative patient characteristics are shown in Table 1. The percentages of patients with digit ratios ≥ 1 and < 1 were 19.1% and 80.9%, respectively. When comparing the baseline factors for digit ratio, there were statistically significant differences in sex and age. In the group with digit ratio ≥ 1, 56.3% were female, and the average age was 59.5 years. In the group with a digit ratio < 1, 41.3% were female, and the average age was 63.4 years (*p* = 0.001 for sex, *p* = 0.01 for age). However, there were no significant differences in motion sickness, nonsmoking, or postoperative opioid use. The number of risk factors in the Apfel simplified risk score did not vary between the two groups. Although anesthesia with propofol was more common in the group with a digit ratio ≥ 1, the number of prophylactic measures for PONV did not differ between the two groups.

**TABLE 1:** Patient characteristics and perioperative information.

	**Digit ratio**		**p** **value**	**SMD**
	**< 1**	**≥ 1**		
*n*	641	151		
Age (y), mean ± SD	63.4 ± 16.3	59.5 ± 17.5	0.01	0.23
Under 50 years (%)	149 (23.2)	48 (31.8)	0.036	0.19
Female (%)	265 (41.3)	85 (56.3)	0.001	0.30
BMI, mean ± SD	24.2 ± 12.7	23.9 ± 4.4	0.81	0.026
ASA‐PS 1 (%)	90 (14.0)	30 (19.9)	0.32	0.16
2 (%)	408 (63.7)	92 (60.9)		
3 (%)	137 (21.4)	28 (18.5)		
4 (%)	6 (0.9)	1 (0.7)		
Number of risk factors 0 (%)	31 (4.8)	6 (4.0)	0.48	0.17
1 (%)	183 (28.5)	38 (25.2)		
2 (%)	256 (39.9)	56 (37.1)		
3 (%)	140 (21.8)	40 (26.5)		
4 (%)	31 (4.8)	11 (7.3)		
Nonsmoking (%)	550 (85.8)	124 (82.1)	0.25	0.10
Postoperative opioid usage (%)	308 (48.0)	67 (44.4)	0.47	0.074
History of PONV or motion sickness (%)	112 (17.5)	36 (23.8)	0.079	0.18
Volatile anesthesia (%)	154 (24.0)	24 (15.9)	0.031	0.20
Duration of anesthesia (hr), mean ± SD	4.1 ± 2.1	3.9 ± 2.1	0.2	0.12
Total fentanyl dose (mg), median (IQR)	0.20 (0.10–0.35)	0.20 (0.10–0.35)	0.90	0.027
Total morphine dose (mg), median (IQR)	0.0 (0.0–0.0)	0.0 (0.0–0.0)	0.57	0.042
Number of preventive measures 0 (%)	19 (3.0)	4 (2.6)	0.42	0.20
1 (%)	40 (6.2)	8 (5.3)		
2 (%)	420 (65.5)	91 (60.3)		
3 (%)	157 (24.5)	48 (31.8)		
4 (%)	5 (0.8)	0 (0.0)		
Propofol usage (%)	486 (75.8)	127 (84.1)	0.03	0.21
Dexamethasone usage (%)	347 (54.1)	83 (55.0)	0.93	0.017
Droperidol usage (%)	96 (15.0)	17 (11.3)	0.30	0.11
Granisetron usage (%)	410 (64.0)	100 (66.2)	0.64	0.047
Ondansetron usage (%)	30 (4.7)	7 (4.6)	1	0.002

*Note:* PONV, postoperative nausea and vomiting; IQR, interquartile range.

Abbreviations: BMI, body mass index; SD, standard deviation.

### 3.2. Primary and Secondary Outcomes

The incidence of PONV within 24 h in the group with digit ratio ≥ 1 and < 1 was 18.5% (28/151) and 18.7% (120/641), respectively. No significant differences were found between the groups (Table 2). Antiemetic drugs were used in 2.6% of patients with digit ratio ≥ 1, and 5.9% for those with digit < 1, with no statistically significant difference found (*p* = 0.155). In the subgroup analyses, the incidence of PONV was examined and stratified by sex, age, and type of surgery (Table 2). No significant difference was found in PONV incidence by digit ratio between sexes. Similarly, among patients aged < 50 years, the difference in PONV incidence by digit ratio was not significant. The incidence of PONV did not differ between the laparoscopic and gynecological surgery groups. However, a higher incidence of nausea and vomiting was observed in patients with a digit ratio ≥ 1 in the abdominal surgery group.

**TABLE 2:** Comparison of PONV incidence based on digit ratio.

	**Digit ratio**		**p** **value**	**SMD**
	**< 1**	**≥ 1**		
Occurrences of PONV (%)	120 (18.7)	28 (18.5)	1	0.005
Antiemetic usage (%)	38 (5.9)	4 (2.6)	0.16	0.16
Women	265	85		
Occurrences of PONV (%)	60 (22.6)	22 (25.9)	0.56	0.076
Antiemetic usage (%)	20 (7.5)	3 (3.5)	0.31	0.18
Men	376	66		
Occurrences of PONV (%)	60 (16.0)	6 (9.1)	0.19	0.21
Antiemetic usage (%)	18 (4.8)	1 (1.5)	0.33	0.19
Under 50 years	149	48		
Occurrences of PONV (%)	23 (15.4)	10 (20.8)	0.38	0.14
Antiemetic usage (%)	4 (2.7)	1 (2.1)	1	0.039
Gynecological	45	25		
Occurrences of PONV (%)	14 (31.1)	10 (40.0)	0.6	0.19
Antiemetic usage (%)	5 (11.1)	1 (4.0)	0.41	0.27
Laparoscopic	153	39		
Occurrences of PONV (%)	37 (24.2)	14 (35.9)	0.16	0.26
Antiemetic usage (%)	10 (6.5)	1 (2.6)	0.47	0.19
Abdominal	193	49		
Occurrences of PONV (%)	47 (24.4)	19 (38.8)	0.049	0.31
Antiemetic usage (%)	17 (8.8)	3 (6.1)	0.77	0.10

*Note:* PONV: postoperative nausea and vomiting.

Abbreviation: SMD, standardized mean differences.

### 3.3. Risk Factors for PONV

Table 3 presents the regression analysis results. In both analyses, the digit ratio was not associated with the incidence of PONV. In the univariable analysis, the four items of the simplified Apfel score, duration of anesthesia, intraoperative fentanyl dose, gynecologic surgery, laparoscopic surgery, and abdominal surgery were significantly associated with a higher incidence of PONV. A multivariable analysis also showed that female (odds ratio [OR] 1.79, 95% confidence interval [CI]: 1.10–2.93, *p* = 0.019), nonsmoking (OR 2.29, 95% CI: 1.14–4.6, *p* = 0.021), history of motion sickness or PONV (OR 2.73, 95% CI: 1.6–4.65, *p* < 0.001), and postoperative opioid usage (OR 1.95, 95% CI: 1.12–3.4, *p* = 0.018) were significantly associated with risk of PONV. In the multivariable model, treating digit ratio as a continuous predictor did not change the findings: The digit ratio was not associated with PONV (adjusted OR per 0.1 increase, 0.61; 95% CI, 0.32–1.16; *p* = 0.131; cohort median 0.960 [IQR 0.940–0.979]).

**TABLE 3:** Univariable and multivariable logistic regression analysis of factors contributing to the incidence of PONV.

	**PONV**		**Univariable**	**p** **value**	**Multivariable**	**p** **value**
	**0**	**1**	**OR**		**OR**	
*n*	644	148				
Digit ratio ≥ 1 (%)	123 (19.1)	28 (18.9)	0.99 (0.63–1.57)	1	0.92 (0.56–1.50)	0.74
Age (y), mean ± SD	62.5 ± 16.7	63.0 ± 16.1	1.00 (0.99–1.01)	0.75	1 (0.99–1.01)	0.81
Under 50 years (%)	164 (25.5)	33 (22.3)	0.84 (0.53–1.30)	0.46	—	—
Female (%)	268 (41.6)	82 (55.4)	1.74 (1.20–2.54)	0.002	1.79 (1.10–2.93)	0.019
Nonsmoking (%)	538 (83.5)	136 (91.9)	2.23 (1.18–4.59)	0.01	2.29 (1.14–4.6)	0.021
Postoperative opioid usage (%)	283 (43.9)	92 (62.2)	2.09 (1.43–3.08)	< 0.001	1.95 (1.12–3.4)	0.018
History of PONV or motion sickness (%)	103 (16.0)	45 (30.4)	2.31 (1.49–3.53)	< 0.001	2.73 (1.6–4.65)	< 0.001
Volatile anesthesia (%)	142 (22.0)	36 (24.3)	1.14 (0.80–1.63)	0.59	1.95 (1.07–3.56)	0.03
Duration of anesthesia (hr), mean ± SD	3.9 ± 2.1	4.5 ± 2.3	1.12 (1.04–1.22)	0.003	1.08 (0.98–1.19)	0.13
Total fentanyl dose (mg), median (IQR)	0.20 (0.10–0.30)	0.25 (0.14–0.40)	2.57 (1.06–6.24)	0.014	0.955 (0.27–3.33)	0.94
Total morphine dose (mg), median (IQR)	0.0 (0.0–0.0)	0.0 (0.0–0.0)	1.07 (0.92–1.25)	0.42	1.05 (0.88–1.24)	0.61
Cholecystectomy (%)	8 (1.2)	4 (2.7)	2.21 (0.48–8.37)	0.25	1.64 (0.44–6.05)	0.46
Gynecological (%)	46 (7.1)	24 (16.2)	2.51 (1.41–4.38)	0.001	1.66 (0.79–3.48)	0.18
Laparoscopic (%)	141 (21.9)	51 (34.5)	1.87 (1.24–2.80)	0.002	1.19 (0.60–2.34)	0.62
Abdominal (%)	175 (27.2)	66 (44.6)	2.14 (1.45–3.14)	< 0.001	1.01 (0.47–2.18)	0.98
Propofol usage (%)	502 (78.0)	111 (75.0)	1.07 (0.73–1.55)	0.45	0.478 (0.26–0.87)	0.015
Dexamethasone usage (%)	331 (51.4)	99 (66.9)	1.85 (1.29–2.66)	0.001	0.91 (0.54–1.54)	0.72
Droperidol usage (%)	93 (14.4)	20 (13.5)	0.92 (0.58–1.47)	0.90	0.44 (0.23–0.88)	0.019
Granisetron usage (%)	398 (61.8)	112 (75.7)	1.92 (1.33–2.79)	0.002	0.91 (0.52–1.59)	0.74
Ondansetron usage (%)	33 (5.1)	4 (2.7)	0.57 (0.20–1.64)	0.28	0.43 (0.13–1.4)	0.16

*Note:* PONV, postoperative nausea and vomiting; IQR, interquartile range.

Abbreviations: OR, odds ratio; SD, standard deviation.

The prophylactic medications for PONV include propofol, dexamethasone, droperidol, granisetron, and ondansetron. In multivariable analysis, propofol (OR 0.48, 95% CI: 0.26–0.87, *p* = 0.015) and droperidol (OR 0.44, 95% CI: 0.23–0.88, *p* = 0.019) were found to significantly reduce the incidence of PONV, while no statistically significant differences were observed for the other medications.

## 4. Discussion

In this prospective observational study, we investigated whether a more feminine digit ratio (≥ 1) independently increases the incidence of PONV following general anesthesia, thereby evaluating its potential as a simple, noninvasive marker that could supplement the Apfel simplified risk score and guide prophylaxis. In the overall cohort, no statistically significant association between digit ratio and PONV was observed. However, among patients undergoing abdominal surgery, a digit ratio ≥ 1 was associated with a higher incidence of PONV. These findings indicate that although digit ratio is not a universal predictor of PONV, it may hold predictive value within select high‐risk populations.

A digit ratio ≥ 1 is considered more feminine and is associated with multiple sex hormones. In this study, because the incidence of PONV might vary with sex hormones, we hypothesized that a digit ratio ≥ 1 might be a risk factor for PONV and conducted a comparative study based on this assumption. As for the digit ratio related to background factors, the percentage of women was different (Table 1), but this was to be expected since previous studies [14, 15] have shown that women are more likely to have a second finger longer than a fourth finger. This suggests that the digit ratio may be an acceptable alternative indicator of sex hormone levels. However, the results of this study suggest that a digit ratio ≥ 1 is not a risk factor for PONV in all eligible patients, and we speculate several explanations. First, the digit ratio is generally regarded as a marker of comparatively stable interindividual differences in the hormonal profile rather than short‐term fluctuations. Because these differences typically emerge over years, as indicated by associations with cancer risk [19], number of pregnancies [17], and age at menopause [16], they are unlikely to be reflected in a short‐term outcome, such as PONV after a single anesthetic exposure. Conversely, transient hormonal changes, particularly in young women, may exert a stronger short‐term influence and obscure any small background effect indexed by the digit ratio. As surgery is a one‐time event, PONV risk is likely driven predominantly by perioperative factors and acute hormonal states, with limited sensitivity to stable interindividual markers, such as the digit ratio. Second, in this study, we used a digit ratio cut‐off of one based on previous research [15–18]. As a larger proportion of women had a digit ratio below one, an alternative cut‐off might arguably be more appropriate. Nevertheless, to avoid arbitrariness and to preserve clinical interpretability and ease of use, we did not deviate from this standard in our research. Moreover, modeling digit ratio on its continuous scale yielded no significant association with PONV, and our overall conclusions were unchanged. Third, differences in anesthesia methods and PONV prevention strategies were observed between digit ratio groups. Propofol anesthesia was significantly more common in the digit ratio ≥ 1 group compared to the digit ratio < 1 group. While the impact may be limited because of the lack of a predominant difference in the total number of cases prevented, the anesthesia method possibly affected the occurrence of PONV. Fourth, informed by prior literature, we considered whether digit ratio and PONV might share genetic determinants. Tentative links between digit ratio and androgen and estrogen receptor biology have been proposed, but the evidence remains inconclusive and often limited by sex composition and other population features [22, 23]. For PONV, genome‐wide and candidate gene studies have suggested associations with specific genes/variants; however, the findings tend to be cohort‐ or population‐specific, with limited generalizability by sex [24, 25]. Multiple reports also implicate the 5‐HT3 receptor pathway and variability in antiemetic response [26, 27], and TACR1 has been proposed as a candidate with a promoter SNP within a putative estrogen response element, suggesting a potential hormone‐responsive mechanism [28]. Nevertheless, we found no consistently shared genes between the digit ratio and PONV in the literature. As our cohort did not include genotyping, we were unable to evaluate this hypothesis. Future studies combining preoperative DNA sampling with detailed perioperative phenotyping may be warranted to examine potential overlap between genetic factors for the digit ratio and for PONV.

There was no association between digit ratio and PONV in any of the cases. However, subgroup analysis indicated that digit ratio may be a risk predictor in patients who underwent abdominal surgery. A recent large multicenter study [21] suggested that abdominal surgery was associated with a risk of PONV. In line with this, our study analyzed a group of patients who underwent abdominal surgery. On comparing the background factors that are risk factors for PONV, such as those used in the multivariable analysis, between the abdominal surgery group and other surgeries, differences were observed in postoperative opioid usage, total fentanyl dose, and duration of surgery. Abdominal surgeries involving the peritoneum are often associated with a higher degree of pain, leading to increased intraoperative and postoperative opioid use, which could increase the risk of PONV. This highlights the association between the digit ratio and the incidence of PONV. Future studies focusing solely on high‐risk groups will show that the digit ratio is an additional risk factor for PONV.

This study has some limitations. First, based on previous studies, we assumed that the digit ratio is related to sex hormones, but we did not measure hormone levels using a blood test. Consequently, this study did not investigate whether there was a relationship between the digit ratio and hormone levels. However, it is not reasonable to perform such tests on all surgical patients, and the actual measured values may change. Therefore, these measurements were not performed in this study. Second, there is a potential difference in the relationship between digit ratio and sex hormone levels between men and women. This raises concerns about the validity of analyzing males and females together in this study. To address this concern, we conducted separate analyses for men and women. However, there were no significant differences in the incidence when comparing the two groups. This finding supports the validity of our initial combined analysis, though this limitation should be taken into account when interpreting the results. While the findings were consistent, further studies are needed to explore these sex‐specific associations in greater detail. This study serves as a valuable foundation for future research.

In conclusion, this study showed that the digit ratio was not associated with PONV across the entire study population; however, it might be useful in subgroup analysis of patients undergoing abdominal surgery. Further studies are required to examine the association between the digit ratio and PONV in a sufficient sample size of patients at high risk for PONV.

**Nomenclature**
PONV: Postoperative nausea and vomiting
ICU: Intensive care unit
OR: Odds ratio
CI: Confidence interval

## Author Contributions

Yoshihiko Chiba conducted the study, including the design, data collection, and analysis. Yoshihiko Chiba also interpreted the data and wrote the manuscript. Yusuke Iizuka helped design the work, interpreted the data, and substantially revised the manuscript. Shinnosuke Ohama and Kyoko Chiba helped with data collection. Koichi Yoshinaga, Kyosuke Takahashi, and Masamitsu Sanui helped design the study and critically revised the manuscript for important intellectual content.

## Funding

The authors have nothing to report.

## Disclosure

All the authors have read and approved the final version of the manuscript.

## Conflicts of Interest

The authors declare no conflicts of interest.

## Data Availability

The data that support the findings of this study are available upon request from the corresponding author. The data are not publicly available due to privacy or ethical restrictions.
